# Construction and Evaluation of Quantitative Small-Animal PET Probabilistic Atlases for [^18^F]FDG and [^18^F]FECT Functional Mapping of the Mouse Brain

**DOI:** 10.1371/journal.pone.0065286

**Published:** 2013-06-07

**Authors:** Cindy Casteels, Kathleen Vunckx, Sarah-Ann Aelvoet, Veerle Baekelandt, Guy Bormans, Koen Van Laere, Michel Koole

**Affiliations:** 1 Division of Nuclear Medicine, Department of Imaging and Pathology, KU Leuven, Leuven, Belgium; 2 MoSAIC – Molecular Small Animal Imaging Centre, KU Leuven, Leuven, Belgium; 3 MIRC – Medical Imaging Research Centre, University Hospitals and KU Leuven, Leuven Belgium; 4 Laboratory for Neurobiology and Gene Therapy, Department of Neurosciences, KU Leuven, Leuven, Belgium; 5 Laboratory for Radiopharmaceutical Chemistry, Department of Pharmaceutical and Pharmacological Sciences, KU Leuven, Leuven, Belgium; University G. D’Annunzio, Italy

## Abstract

Automated voxel-based or pre-defined volume-of-interest (VOI) analysis of small-animal PET data in mice is necessary for optimal information usage as the number of available resolution elements is limited. We have mapped metabolic ([^18^F]FDG) and dopamine transporter ([^18^F]FECT) small-animal PET data onto a 3D Magnetic Resonance Microscopy (MRM) mouse brain template and aligned them in space to the Paxinos co-ordinate system. In this way, ligand-specific templates for sensitive analysis and accurate anatomical localization were created. Next, using a pre-defined VOI approach, test-retest and intersubject variability of various quantification methods were evaluated. Also, the feasibility of mouse brain statistical parametric mapping (SPM) was explored for [^18^F]FDG and [^18^F]FECT imaging of 6-hydroxydopamine-lesioned (6-OHDA) mice.

**Methods:**

Twenty-three adult C57BL6 mice were scanned with [^18^F]FDG and [^18^F]FECT. Registrations and affine spatial normalizations were performed using SPM8. [^18^F]FDG data were quantified using (1) an image-derived-input function obtained from the liver (cMR_glc_), using (2) standardized uptake values (SUV_glc_) corrected for blood glucose levels and by (3) normalizing counts to the whole-brain uptake. Parametric [^18^F]FECT binding images were constructed by reference to the cerebellum. Registration accuracy was determined using random simulated misalignments and vectorial mismatch determination.

**Results:**

Registration accuracy was between 0.21–1.11 mm. Regional intersubject variabilities of cMR_glc_ ranged from 15.4% to 19.2%, while test-retest values were between 5.0% and 13.0%. For [^18^F]FECT uptake in the caudate-putamen, these values were 13.0% and 10.3%, respectively. Regional values of cMR_glc_ positively correlated to SUV_glc_ measured within the 45–60 min time frame (spearman r = 0.71). Next, SPM analysis of 6-OHDA-lesioned mice showed hypometabolism in the bilateral caudate-putamen and cerebellum, and an unilateral striatal decrease in DAT availability.

**Conclusion:**

MRM-based small-animal PET templates facilitate accurate assessment and spatial localization of mouse brain function using VOI or voxel-based analysis. Regional intersubject- and test-retest variations indicate that for these targets accuracy comparable to humans can be achieved.

## Introduction

Neuroimaging studies are increasingly performed on mice as models for a variety of human diseases and genetic traits. Transgenic manipulation of mice has created phenotypes that link specific genes to molecular functions in diseased conditions. Mouse models of brain diseases encompass all major neurodegenerative diseases, stroke, but also psychiatric diseases such as depression and anxiety (for review see [1], [2]). Functional small-animal PET imaging allows longitudinal follow-up, which is important when investigating neuropharmacological interventions or disease characterization. As in small-animal PET measurements of mouse brain, the number of available resolution elements is lower than in the human PET counterpart [3], optimal information usage is imperative for detailed regional analysis.

An important step in functional neuroimaging analysis is the development of methods to combine data from different subjects in a common atlas space [4]. Spatial normalization can increase sensitivity of low-magnitude responses and facilitate group comparisons. This can be performed using either automated voxel-based or operator-independent volume-of-interest (VOI) analysis on probabilistic atlases which are oriented into a common stereotactic space, e.g. according to Paxinos for rodents [5].

For small-animal imaging of mice, despite the high demand, digital mouse brain atlases are still sparsely available. With the majority in book-form and histology-based, currently available mouse brain atlases vary in their formats (book or digital), original data source (histology or magnetic resonance imaging (MRI)), sample number, and structure labeling methods (text annotated and structure segmented) [6]–[13]. Besides, digital mouse brain atlases based on PET are still lacking, as well as their alignment in Paxinos stereotactic space. Also, the extension to automated whole-brain analysis methods, either by whole-brain pre-defined volume-of-interest (VOI) analysis or by statistical parametric mapping (SPM) has not been systematically investigated in this species. Especially in the latter case, voxel-based analysis may offer a unique way of extracting information in a fully automated data-driven approach, which has been shown to be superior in detecting pathological signals in humans [14].

In the current study, we have aligned in space an existing high-resolution Magnetic Resonance Microscopy (MRM) mouse atlas [6] to the Paxinos co-ordinate system, onto which we have then mapped functional metabolic ([^18^F]-fluorodeoxyglucose; [^18^F]FDG) and dopamine transporter (DAT using [^18^F]FECT) small-animal PET data of healthy wild-type mouse brains, in order to create ligand-specific templates for assessment of metabolism and dopaminergic neurotransmission. Upon construction of these functional templates, we subsequently assessed the feasibility of operator-independent pre-defined VOI analysis for the detection and quantification of changes in mouse brain, by determining regional test-retest and intersubject variability, as well as right-to-left asymmetry indices for various quantification methods of [^18^F]FDG and [^18^F]FECT. Next, we also evaluated their feasibility in detailed regional SPM analysis of the mouse brain and in reporting findings directly corresponding to the Paxinos co-ordinate system. This was explored in a model of Parkinson’s disease (PD), the 6-hydroxydopamine (6-OHDA)-lesioned mouse model [15] by studying mouse brains in comparison to a control group with [^18^F]FDG and [^18^F]FECT. Finally, the registration accuracy of our automated approach was assessed.

## Materials and Methods

### Ethic Statement

The research protocol was approved by the Committee on the Ethics of Animal Experiments of the University of Leuven (Permit-number: 053/2009) and was according to European Ethics Committee guidelines (decree 86/609/EEC). Institutional guidelines for animal welfare and experimental conduct were followed.

### Animals

In total, experiments were conducted on twenty-three male C57BL6 mice. Animal weight was 22.9±3.4 g at the start of the experiment. All animals were housed in cages of five with an average room temperature of 22°C and a 12-h light/dark cycle. Food and water were given ad libitum.

To demonstrate the feasibility of SPM in using these functional templates, we additionally studied 3 6-OHDA-lesioned mice (age, 10 weeks; body weight range, 18–24 g) 1–3 weeks after creating the lesions. Dopaminergic lesions were created by a unilateral microinfusion of the dopaminergic toxin 6-OHDA into the right striatum using the following stereotaxic co-ordinates according to the mouse brain Paxinos atlas [7]: AP+0.4 mm, ML −1.8 mm and DV −3.5 mm relative to Bregma. The 6-OHDA was dissolved in saline containing 0.1% ascorbic acid at concentrations of 2 µg/µL to create a severe lesion. Two microliters of the 6-OHDA-solution was injected at a rate of 0.25 µL/min using a Hamilton syringe. The needle was left in place for an additional 5 min before being withdrawn to allow diffusion of the 6-OHDA solution.

### Radiotracer Preparation and Imaging

Functional images of the striatal dopamine transporter (DAT) were obtained in each mouse using the radioligand [^18^F]FECT (2′-[^18^F]fluoroethyl (1R-2-exo-3-exe)-8-methyl-3-(4-chlorophenyl)-8-azabicyclo[3.2.1]-octane-2-carboxylate), while metabolic images were obtained using [^18^F]FDG. The synthesis of [^18^F]FECT PET radiotracer was performed according to the procedure by Wilson et al. but using 2-^18^F-fluoroethyltrifluoromethanesulfonate instead of 2-^18^F-fluoroethylbromide [16]. [^18^F]FDG was prepared by using an IBA (Ion Beam Applications, Louvain-la-Neuve, Belgium) [^18^F]FDG synthesis module. Before being imaged, mice were anesthetised with an intraperitoneal injection of a ketamine (75 mg/kg ip., Ketalar®, Pfizer, Belgium) - medetomidine mixture (1.0 mg/kg, Dormitor®, Pfizer). The ketamine-medotomidine mixture was chosen over isoflurane anaesthesia to avoid from the latter possible confounding effects of dosage on [^18^F]FECT binding to the DAT [17]. On average 12.4±1.2 MBq of each radioligand (specific activity range: 100–760 GBq/µmol) was injected into the tail vein using an infusion needle set. The radioligands were diluted with saline to obtain a 5% ethanol solution and injected in a total volume of approximately 300µL. Body temperatures were maintained between 36.5 and 37°C with a heating path during acquisition. After overnight fasting, [^18^F]FDG acquisitions were performed dynamically for 90 min. [^18^F]FECT measurements were acquired until 120 min post injection.

Small-animal PET imaging was performed using an LSO detector-based FOCUS 220 tomograph (Siemens/Concorde Microsystems, Knoxville, TN), which has a transaxial resolution of 1.35 mm full-width at half-maximum (FWHM). Data were acquired in a 128×128×95 matrix with a pixel width of 0.475 mm and a slice thickness of 0.815 mm. Acquisition data were Fourier rebinned in 24 and 27 frames (4×15, 4×60, 5×180, 8×300, 6×600), depending on total scan duration. Sinograms were reconstructed using filtered backprojection (FBP; ramp filter cutoff: 0.5) to avoid possible positive bias due to iterative reconstruction methods [18]. No corrections were made for attenuation or scatter, due to the much smaller size of the mouse head and the lower thickness and density of the skull as compared to primates.

**Figure 1:**
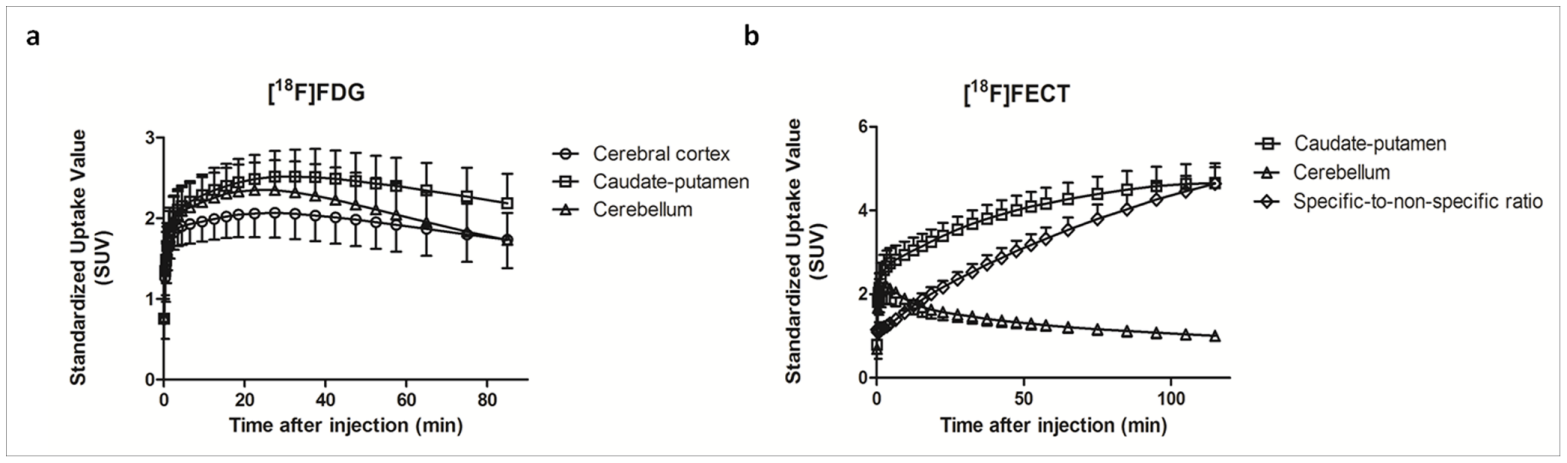
Average time-activity curves for [^18^F]FDG (a) and [^18^F]FECT (b), expressing uptake in the cerebral cortex (○), caudate-putamen (□) and cerebellum (Δ). For [^18^F]FECT, specific-to-non-specific ratio (caudate-putamen to cerebellum (◊)) is also shown.

Time-activity curves of the brain uptake of [^18^F]FDG and [^18^F]FECT are shown in Fig. 1.

A number of control animals were scanned twice to estimate test-retest variability for [^18^F]FECT and [^18^F]FDG ([^18^F]FECT: n = 5; [^18^F]FDG n = 5). The time interval between both scans was 7 days for [^18^F]FECT and 8 days for [^18^F]FDG.

### Atlas Construction in Paxinos Space

**Figure 2:**
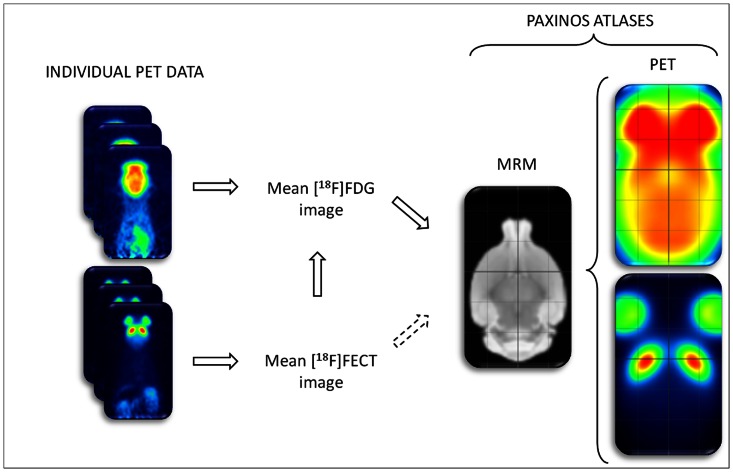
Schematic representation of the functional PET atlases construction pathway. The full arrows express the followed procedure, the arrows in dotted line the objective. On the standardized atlases a stereotactic grid is shown (2 mm interlines).

**Figure 3:**
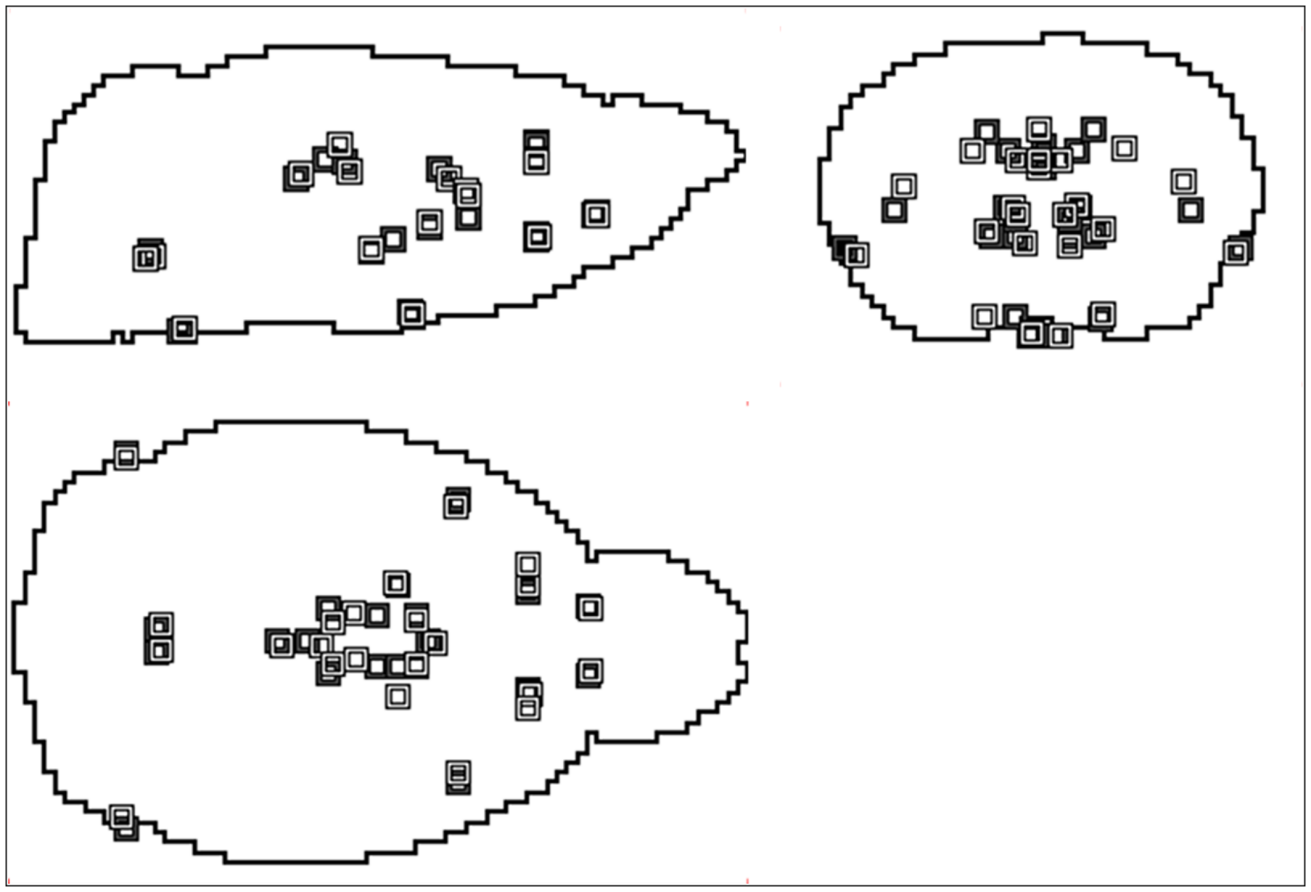
Spatial correspondence achieved between the anatomical landmarks defined in the MRM image and the corresponding co-ordinates in Paxinos space. Points marked with *open symbols* signify the MRM space, whereas points denoted with *full symbols* belong to the Paxinos space.

The flowchart of the procedure for atlas creation is shown in Fig. 2. The high-resolution mouse MRM atlas of the University of California, Los Angeles was used as starting image (http://map.loni.ucla.edu/atlas) [6]. We based the alignment of the mouse MRM atlas space and the Paxinos atlas space [7] upon a label-based affine transformation. Homologous anatomical features in the two image spaces were identified and used to find the affine transformation that best superimposed the labelled points by minimizing the Euclidean distance between all point pairs. To obtain accurate estimates of the transformation parameters, we aimed at identifying as many reliable anatomical structures as possible. In addition, we sought for an even spatial spread of the selected points across the mouse brain volume. In total, twenty-three anatomical landmarks were identified on the high-resolution mouse MRM atlas. All landmarks were previously rated as very to extremely well defined based on their visibility and clearness [19]. The resulting degree of correspondence between the two point sets is demonstrated in Fig. 3.

After realignment, the mouse MRM image volume was re-sampled into a volume that encompassed the spatial extent of the Paxinos atlas. To this end, we used a bounding box of –5 to 5 mm in the x-direction, −10 to 7 mm in the y-direction and –8 to 1 mm in the z-direction. The voxel size was set to 0.2×0.2×0.2 mm, resulting in a volume of 50×85×45 voxels. We selected the bregma as zero-reference plane and arbitrarily defined the x-axis in the MRM template to be positive to the left of the mid-line and to be negative to the right.

For PET atlas reconstruction, the following within-modality procedure was repeated for all individual [^18^F]FDG and [^18^F]FECT images of control mice (n = 10/radioligand). Of the individual PET images, one was selected as the ‘representative’ brain. Each individual PET scan was then transformed into the space of the ‘representative’ scan. The normalized image datasets were averaged voxelwise to create functional image atlases.

Cross-modality ([^18^F]FDG-PET:MRM and [^18^F]FECT-PET:MRM) atlas registrations were performed in order to obtain ligand-specific templates mapped in Paxinos space. The within-modality and cross-modality registrations were performed using 12-parameters affine transformations in SPM8 (Wellcome Department of Cognitive Neurology, London, UK; http://www.fil.ion.ucl.ac.uk/spm/) as this provided the best results on a visual basis. Consequently, a mouse brain spatially normalized to these functional templates will facilitate reporting results in co-ordinates directly corresponding to the Paxinos co-ordinate system [7].

A VOI map, previously defined on the starting image [6], was subsequently also transferred to Paxinos space using the previously determined optimal affine transformation. For this purpose, a binary mask of each VOI was generated, realigned appropriately to Paxinos co-ordinates and re-sampled into the volume that encompassed the spatial extent of the Paxinos atlas using trilinear interpolation. In this way, a label number between 0 and 1 was generated for each voxel and each voxel was then assigned to the VOI corresponding to the highest label.

### Image Processing and Quantification of [^18^F]FDG and [^18^F]FECT

Individual [^18^F]FDG and [^18^F]FECT PET images were normalized to the custom-made, ligand-specific mouse templates in Paxinos stereotactic space, allowing use of the pre-defined VOI map and assessment of its feasibility in quantifying mouse brain data by determining test-retest and intersubject variability.

Time-activity curves were generated for the bilateral caudate-putamen, cerebral cortex and cerebellum of each individual scan using PMODv.3.1 (PMOD Inc, Zurich, Switzerland). For time-activity curve generation, we have used macrostructures larger than 9 mm^3^, since it has previously been showed that this size avoids partial volume effects on a small-animal PET system similar to ours [20].

The following methods of quantification were evaluated for [^18^F]-FDG: (i) using an image derived input function obtained from the liver, (ii) using blood samples to quantify standardized uptake values (SUV), and (iii) normalizing counts to the whole-brain uptake. For [^18^F]FECT data, voxelwise parametric DAT binding images (BP) were constructed from original FBP data by reference to the cerebellum using the Ichise MRTM2 module in PMOD [21].

The use of the liver time-activity curve as a surrogate input of [^18^F]FDG has extensively been validated previously [22], as well as by us in a limited number of subjects prior to study set-up (Figure S1). The liver is useful as an input since it is a large blood pool with relatively low [^18^F]FDG retention. We have chosen the liver time-activity curve over the heart time-activity curve as a surrogate input to avoid spillover from the surrounding myocardium.

In line with [22], we converted the liver time-activity curve to an input function using the following equation: R_FDG = _0.39*e* ^−0.19t^+1.17, where R_FDG_ is the ratio of plasma to whole blood as a function of blood-sampling time (in minutes) after tracer injection. The [^18^F]FDG uptake constant (K*_FDG_) and the cerebral glucose metabolic rate (cMR_glc_) were then calculated using a 3-compartment model by 
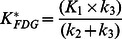
 and 
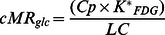
, respectively, where LC is a lumped constant representing the ratio of [^18^F]FDG utilization to actual glucose utilization in the brain and *Cp* is the glucose concentration measured in the blood before small-animal PET imaging. A LC of 0.6 was used in this study as all animals were within the 4.01 to 14.75 mmol/l range [22]–[24]. Constant glucose concentration is assumed in the kinetic analysis and was evaluated in a subset of animals (n = 5). The amount of changes in blood glucose concentration of 90 min was approximately −11.8% (SD 0.20; NS).

SUVs were calculated from the 45–60 min time interval with and without correction of blood glucose, as previously described [25], [26]. The following equations were used: 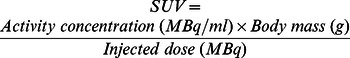 and 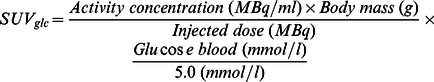.

Note that the measured glucose content is normalized for an overall population average of 5.0 mmol/l so that the SUVs with and without correction of glucose content are numerically practically identical (on average) [27]. In this study, cMR_glc_ values using the 3-compartment model were considered as the standard and compared to the various other methods of quantification of [^18^F]FDG.

### Statistical Parametric Mapping

Upon spatial normalization of individual images to the ligand-specific mouse templates, also a categorical subject design using conditions (6-OHDA vs. wild-types) was carried out on parametric DAT and [^18^F]FDG images of three 6-OHDA-lesioned mice in comparison to the respective control data. SPM analysis was performed without global normalization, with a threshold of 0.8 of the maximum image intensity. For statistical analysis, T-maps data were interrogated at a p_height_ level of p = 0.005 (uncorrected) and extent threshold k_ext_ >200 voxels. Only clusters with p<0.05 corrected were withheld as significant.

### Determination of Registration Accuracy

The following experiment was performed to quantitatively assess the registration accuracy of mapping individual data to the ligand-specific PET templates, similar to previously described [28]. Simulated [^18^F]FDG and [^18^F]FECT PET data, derived from the pre-defined VOI map and representing a control mouse and a 6-OHDA-lesioned animal, underwent each time 40 random misalignments: 10 translations, 10 rotations, 10 linear stretching and 10 combinations of the three previous parameters. The misalignments were uniformly distributed (random number generation function in Excel 2007, Microsoft Inc, USA) within −5 to +5 mm of translation, −20° to +20° of rotation, −10% to +10% scaling along the three orthogonal axes/planes. For the combination misalignments, rotations were only allowed within the range of 10 degrees in the three main planes. The amount of deliberate misalignment was based on typical magnitudes that can occur in realistic situations. Each resultant image volume was then reregistered to the study-specific template using the automated spatial normalization procedure (12 parameter affine) integrated in SPM8. For each voxel (x,y,z) in the original image, the position (x+*Δ*_x_, y+*Δ*_y_, z+*Δ*_z_) after re-registration was computed. This position was determined by means of 3 self-constructed images in which neighbouring voxels differ 1 unit, in the x, y and z-direction, respectively. The distance (*Δ*_x_^2^+*Δ*_y_^2^+*Δ*_z_^2^)^1/2^ was averaged over all mouse brain voxels and used as a measure of registration accuracy.

### General Statistics

Regional intersubject and test-retest variability as well as right-to-left asymmetry indices were calculated from VOI-derived measurements of radioactivity concentration. The VOIs used in the current study were larger than 9 mm^3^, as a recent study on a small-animal PET system similar to ours [29], showed that this size avoids partial volume effects. The test-retest variability for radioligand index R_i_ in VOI region i was determined as : |R_i,1_–R_ i,2_|*2/(R_ i,1_+ R_ i,2_), where the indices 1 and 2 refer to the two different scans of the same animal.

Reported values are given as the mean±SD. Conventional statistics were carried out using GraphPad Prism v5.0 (San Diego, CA, USA). Significance was defined at the 95% probability level.

## Results

### [^18^F]FDG and [^18^F]FECT Probabilistic Atlases in Paxinos Co-ordinate Space

**Figure 4:**
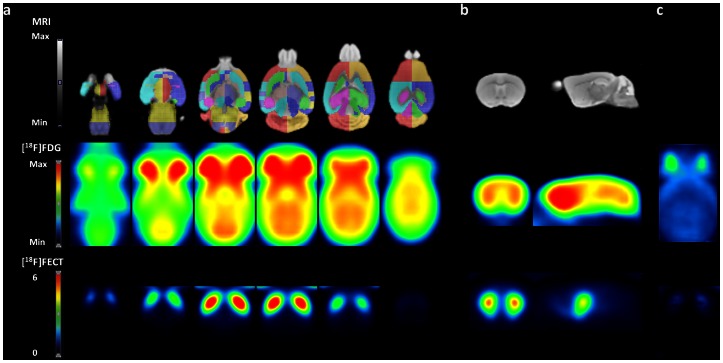
[^18^F]FDG and [^18^F]FECT probabilistic atlases. (a) Series of axial sections (ventral to dorsal, slice interval 1.2 mm, radiological orientated) through the mouse brain MRM atlas (top row) and co-registered [^18^F]FDG (middle row) and [^18^F]FECT (bottom row) small-animal PET template. (b) One representative coronal (radiological orientation) and sagittal slice through caudate-putamen. (c) Horizontal section through variance map at level z = −2.8 (relative to bregma). The mouse brain MRM atlas shows the pre-defined VOI map over-layed. Color bars indicate relative intensities for [^18^F]FDG; binding potential for [^18^F]FECT.

Fig. 4 shows the glucose metabolic and DAT small-animal PET templates aligned in space to the mouse brain atlas of Paxinos. The regional mean uptake, intersubject and test-retest variability as well as right-to-left asymmetry indices of [^18^F]FDG and [^18^F]FECT are displayed in Table 1.

**Table 1:** Mean [^18^F]FDG and [^18^F]FECT uptake, intersubject variability, test-retest and right-to-left asymmetry indices obtained using pre-defined VOI-analysis.

[^18^F]FDG	Mean	SD	Inter -Subject	Test - Retest	Ri/Le Ratio	SD	Volume
*Cerebral metabolic rate of glucose (cMR_glc_)*							
Cerebral Cortex	44.49	6.86	15.41	5.02	1.00	0.02	0.131
Caudate-putamen	52.01	9.73	18.71	7.06	0.98	0.03	0.021
Cerebellum	55.82	10.73	19.23	13.00	1.04	0.04	0.048
*Standardized uptake value (SUV)*							
Cerebral Cortex	1.95	0.33	16.92	27.74	1.03	0.04	–
Caudate-putamen	2.43	0.35	14.26	24.87	1.00	0.02	–
Cerebellum	2.11	0.39	18.47	29.16	1.00	0.03	–
*Standardized uptake value corrected for blood glucose (SUV_glc_)*							
Cerebral Cortex	1.80	0.28	15.78	11.11	–	–	–
Caudate-putamen	2.25	0.34	15.29	10.99	–	–	–
Cerebellum	1.95	0.34	17.49	11.00	–	–	–
*Whole-brain normalized uptake*							
Cerebral Cortex	0.90	0.09	9.92	5.73	–	–	–
Caudate-putamen	1.13	0.09	7.59	2.81	–	–	–
Cerebellum	0.97	0.07	7.68	3.57	–	–	–
**[^18^F]FECT**	**Mean**	**SD**	**Inter -Subject**	**Test - Retest**	**Ri/Le Ratio**	**SD**	**Volume**
*Binding Potential (BP)*							
Caudate-putamen	4.14	0.54	13.05	10.32	1.06	0.03	0.021

Intersubject (n = 10) and test-retest (n = 5) coefficients of variation are expressed as percentages; the volumes of the VOIs are expressed in cm^3^; SD: standard deviation.

As can be seen from these templates and Table 1, values of cMR_glc_ in the mouse brain show a fairly symmetrical uptake in the cortex, caudate-putamen and highest relative activity in the cerebellum, which is comparable to the [^18^F]FDG distribution pattern in rats [28]. Intersubject variation for [^18^F]FDG ranged from 15.4% in the cerebral cortex to a maximum value of 19.2% in the cerebellum. Regional test-retest values for metabolic data were slightly lower with a range from 5.0% in the cerebral cortex to 13.0% in the cerebellum. Right-to-left ratio ranged from 0.98 (±0.03) in the caudate-putamen to 1.04 (±0.04) in the cerebellum.

For [^18^F]FECT, caudate-putamen binding was on average 4.14 with a standard deviation of 0.54, which is lower than in rats, probably due to partial volume effect. In rats, a binding index of almost 9 was found using the same radioligand [28]. The test-retest variability of the caudate-putamen was of the same order as intersubject variability, namely 10.3% and 13.1% respectively.

### Comparison of Regional cMR_glc_ to SUV, SUV_glc_ and Whole-brain Normalized [^18^F]FDG

**Figure 5:**
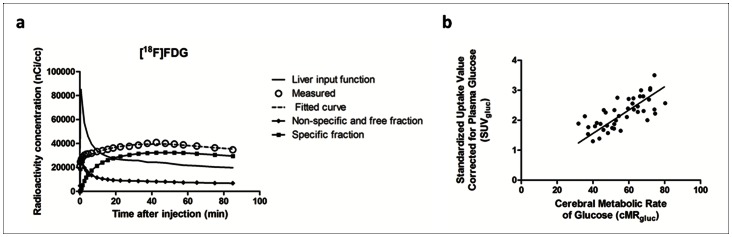
(a) Representative fit of the cortical [^18^F]FDG time-activity-curve using a 3-compartment model *(dashed line)* and liver input function *(solid line)*. (b) Comparison of regional values of cMR_glc_ derived from a 3-compartment model fit to SUV_glc_ measured between 45 min and 60 min post injection. *Solid line* linear regression; spearman r = 0.73; p<0.0001.

The fitting of the cortical, cerebellar and caudate-putamen time-activity curves by the 3-compartement [^18^F]FDG model upon ketamine anesthesia was good in all subjects (R^2^ = 0.93±0.08; n = 10). Figure 5a shows a representative example of the cortical [^18^F]FDG kinetic analysis. Table 2 describes regional K*_FDG_, K_1_/k_2_, k_3_ and k_4_ estimates obtained from ketamine-anesthetized mice scanned over 90 min. The K*_FDG_, K_1_/k_2_, k_3_ and k_4_ values of the cerebral cortex are 0.057 mL/min/g, 0.326 mL/g, 0.183 min^−1^ and 0.044 min^−1^, respectively. Estimates of cMR_glc_ by the 3-compartment model are 44.5±6.9 µmol/min/100g for the cerebral cortex, 52.0±9.7 µmol/min/100g for the caudate-putamen and 55.8±10.7 µmol/min/100g for the cerebellum.

**Table 2:** Regional K*_FDG_, K_1_/k_2_, k_3_ and k_4_ estimates of [^18^F]FDG obtained from the mice scanned over 90 min using a 3-compartment model and liver input function.

Region	K*_FDG_	K_1_/k_2_	k_3_	k_4_
Cortex	0.057 [0.049–0.070]	0.326 [0.273–0.371]	0.183 [0.149–0.252]	0.044 [0.034–0.053]
Caudate-putamen	0.067 [0.055–0.087]	0.366 [0.288–0.421]	0.194 [0.147–0.308]	0.039 [0.032–0.047]
Cerebellum	0.072 [0.058–0.095]	0.341 [0.261–0.425]	0.224 [0.150–0.321]	0.052 [0.038–0.063]

Data are expressed as mean [min-max]; n = 10 K*_FDG_, uptake constant (ml/min/g); K_1_, uptake rate constant (ml cm^−3^ min^−1^); k_2_, clearance rate constant (min^−1^); k_3_, phosphorylation rate constant (min^−1^); k_4_, dephosphorylation rate constant (min^−1^); min, minimum; max, maximum.

When comparing cMR_glc_ to the use of standardized uptake values to simplify [^18^F]FDG quantification, regional intersubject variabilities of SUV and SUV_glc_ were on average comparable to those of cMR_glc_, i.e. ∼16.5% and ∼16.2%, respectively. In contrast, regional SUV values of test-retest (∼27%) were much higher in comparison to SUV_glc_ (∼11%) and cMR_glc_ (∼8%). The test-retest variability of whole-brain normalized data was slightly lower than its intersubject variability, namely ∼4.0% and ∼8.4% respectively.

Regional values of cMR_glc_ positively correlated to SUV_glc_ measured within the 45–60 min time frame (spearman r = 0.71, p<0.0001; Fig. 5b). No correlations of cMR_glc_ with SUV and whole-brain normalized data were found.

### SPM Analysis of 6-OHDA-lesioned Mouse Model for Parkinson’s Disease

**Figure 6:**
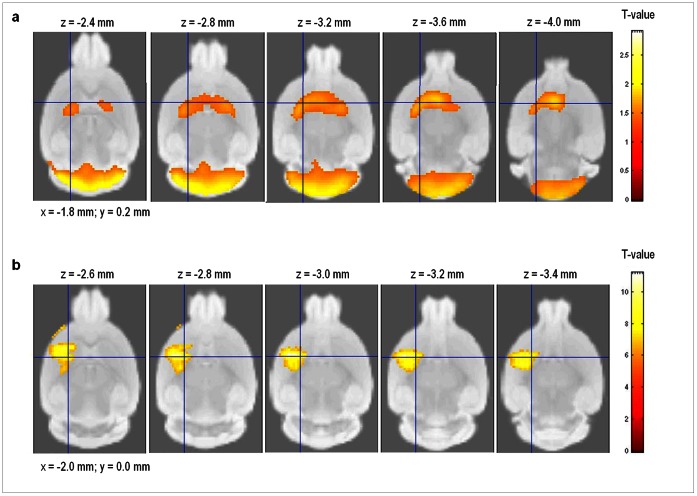
Statistical parametric maps of 6-OHDA mice. Differences for the brain-regions have been color-coded and are superimposed on the MRM template. Series of axial sections with t-maps rendered on the MRM atlas of the mouse brain show significant reductions in glucose metabolism (a) and DAT availability (b). The colored bars on the right express T-score levels. The intersection points of the axial planes have been set to the position of the right caudate-putamen, i.e. (x,y) = (−1.8, 0.2) and (x,y) = (−2.0, 0.0) for [^18^F]FDG and [^18^F]FECT, respectively. Images are in radiological convention.

Statistical parametric maps from the metabolic/presynaptic dopaminergic characterization of 6-OHDA-lesioned mice, compared to wild-type animals are shown in Fig. 6. A significant glucose hypometabolism was seen in comparison to controls in the bilateral caudate-putamen (p-value peak maximum = 0.005, T = 2.90; K_ext_ = 2077; Paxinos co-ordinate peak maximum (x,y,z) = (−1.8, −0.2, −3.6) and bilateral cerebellum (p-value peak maximum = 0.003, T = 3.49; K_ext_ = 4020; Paxinos co-ordinate peak maximum (x,y,z) = (−1.6, −6.6, −1.8)). The mean caudate-putamen and cerebellar glucose metabolic decrease (% change vs. controls) was −33.6±13.5% and −28.5±4.3%, respectively.

Also, a highly significant reduction in DAT availability of the right caudate-putamen was noted in lesioned mice, compared to controls (p-value peak maximum = 1.9 10^−6^, T = 11.17; K_ext_ = 1189). The mean intensity of the deficit (% change in caudate-putamen BP_ND_ vs. controls) was −200.0±16.6% at the Paxinos co-ordinate peak maximum (x,y,z) = (−2.0, 0.0, −3.0).

### Registration Accuracy

Mean registration errors obtained when comparing guided misaligned simulated PET image volumes of a control and a 6-OHDA-lesioned animal to its original spatially normalized image volume are summarized in Table 3. All results are reported in mm.

**Table 3:** Estimates of registration accuracy for a simulated [^18^F]FDG and [^18^F]FECT image of a control and a 6-OHDA-lesioned animal, derived from the VOI map, after re-registration of randomly misaligned data.

	[^18^F]FDG		[^18^F]FECT	
*CONTROL*	Mean	Range	Mean	Range
Translation	0.301	0.180–0.432	0.994	0.211–1.680
Rotation	0.389	0.206–0.608	0.977	0.132–1.727
Linear Stretching	0.452	0.128–0.847	0.978	0.088–1.864
Combined	0.591	0.173–1.112	1.111	0.336–2.002
***6-OHDA-LESIONED ANIMAL***	**Mean**	**Range**	**Mean**	**Range**
Translation	0.283	0.159–0.417	1.011	0.569–1.400
Rotation	0.291	0.160–0.436	1.055	0.292–1.758
Linear Stretching	0.451	0.096–0.863	1.085	0.449–1.806
Combined	0.473	0.106–0.907	1.044	0.393–1.750

All values are expressed in mm.

For the control condition, in all cases, best results were obtained for [^18^F]FDG data (0.301–0.591 mm), while [^18^F]FECT data showed the largest registration errors, likely due to the absence of very specific signal outside the striatum (0.977–1.111 mm). Best results were also obtained when the misalignments were based on translations and rotations only, while combined transformations resulted in the largest registration errors.

For the 6-OHDA-lesioned data, re-registration of randomly misaligned simulated PET data to the ligand-specific templates was on average comparable to the control data, despite the induced lesion and the corresponding functional alterations, which result in asymmetric image information. Also here, the largest registration error was obtained for [^18^F]FECT (1.044–1.085 mm) as compared to [^18^F]FDG (0.283–0.473 mm). Overall, registration accuracy was between 0.21–0.35 of the 1.35 mm FWHM for [^18^F]FDG and between 0.72–0.82 of the 1.35 mm FWHM for [^18^F]FECT.

## Discussion

This work describes the construction of MRM-based functional mouse brain PET templates, aligned in space to the Paxinos co-ordinate system, and evaluates their use in the automated standardized analysis of functional data by using a pre-defined VOI map or statistical parametric mapping. In this work, the intersubject and test-retest variability, as well as the registration errors obtained indicate that accuracy can be achieved which is comparable to the human situation.

The total number of resolution elements in the mouse brain (weighting about 0.3–0.4 g) is limited to a few hundred calculated from a resolution of 1.35 mm FWHM. It is therefore of utmost importance that the amount of information is used maximally in these functional images. The limitations of operator-dependent ROI or VOI approaches are well known [14]. The optimal way to maximize performance is by using a data-driven approach such as statistical parametric mapping, while in some instances for reasons of a priori hypotheses or for sensitivity reasons, a pre-defined VOI approach may be preferred. In both cases, added variability through inter- and intra-observer subjectivity in placement of regions is eliminated, lowering the variability in PET measurements across studies and institutions. As a consequence, a more accurate, sensitive and objective analysis is possible, also enhancing the consistency of image interpretation, independent of one’s experience. In our approach, the use of MRM-based functional mouse brain PET templates in Paxinos co-ordinate space allows analyses on VOI and voxel level to be performed with minimal end-user interaction, as well as the interpretation of our findings to be translation-able to humans.

Using above functional templates, various methods of quantification were evaluated to assess their usefulness in the detection and quantification of changes in mouse brain. Using a 3-compartment model with input function derived from the liver for [^18^F]FDG quantification, we obtained values of K*_FDG_ and cMR_glc_ similar to previous work using the same approach in mice [22]. Our kinetic data also favoured a nonzero k_4_. According to Gosh et al., there is substantial expression of functional glucose-6-phosphatase-β and glucose-6-phospate transporter in mouse astrocytes that accounts for more than 50% of the cell mass, and may thus be responsible for the molecular basis of a non-negligible k_4_ [30]. This fractional rate of dephosphorylation becomes increasingly important as time proceeds.

Although the input function derived from the liver is not purely arterial input, the liver itself is a large blood reservoir - approximately 25% of the liver volume is accounted for blood volume, and had low [^18^F]FDG uptake, which favours its use as a surrogate input function, as recently validated [22]. In the present work, we have also chosen the liver time-activity curve as an surrogate input over an image-derived heart time-activity curve to avoid spillover from the surrounding myocardium [23]–[25]. Besides, it is difficult in mice to obtain spatial-invariant time-activity curves of the heart due to respiratory and heart motions. Other sources of image-derived input functions could potentially include the carotid artery, which is not biased by extremely active surrounding muscle, although resolution items probably limit its use as well.

Using the cMR_glc_ values with liver input function as standard references, we showed that SUV_glc_ measured within the 45–60 min time frame positively correlated to regional values of cMR_glc_ (spearman r = 0.71). Although the correction for blood glucose level in the SUV formula has until now been controversial [31], [32], a comparable finding has been observed by Wong and coworkers [24], who showed that SUV declines with increasing blood glucose level in mice_._ Also, Schiffer *et al.*, stated that a single blood point should be used for SUV quantification in rats to yield similar values of MR_glc_ calculated using the Patlak method [23]. In the present study, correcting SUV for blood glucose level resulted also in a decrease of the regional test-retest coefficients of variation, comparable to values of cMR_glc_. Therefore, results based on SUV should preferably be corrected for blood glucose level and interpreted cautiously, since they may not necessary reflect cMR_glc_ in the tissue of concern. We used levels of blood glucose measured before the small-animal PET as it remained constant during the acquisition.

The obtained test-retest coefficients of variation of cMR_glc_ and SUV_glc_ for [^18^F]FDG, and of BP for [^18^F]FECT are in magnitude consistent with literature measurements in humans [33], [34] and in animal studies using [^18^F]FDG and [^18^F]FECT small-animal PET [28], [35]. This confirms the possible utility of small-animal imaging in mice as a molecular biomarker tool for longitudinal evaluation e.g. in studies of disease progression or after neuromodulatory interventions.

In the present work, we have also demonstrated the feasibility of these functional mouse brain templates in reporting SPM results directly corresponding to the Paxinos co-ordinate system. SPM analysis of [^18^F]FDG and [^18^F]FECT data in 6-OHDA-lesioned mice and wild-type animals pointed towards a hypometabolism in the bilateral caudate-putamen and cerebellum, and towards a unilateral striatal decrease in DAT availability. The unilateral striatal reduction in DAT of 6-OHDA mice has been demonstrated using the DAT radioligand [^11^C]methylphenidate [15]. Our Paxinos co-ordinate peak maximum was (x,y,z) = (−2.0, 0.0, −3.0) and the SPM cluster co-ordinates encompassed the entire unilateral caudate-putamen (z co-ordinate range, −2.6 to −3.4), demonstrating feasibility of our approach.

Validating the registration accuracy of its use is of vital importance, but often difficult. Determining registration errors by observer assessment and external fiducial markers has some drawbacks. From our experience, reproducibility of animal positioning within a restraining or even stereotactic device proves to be insufficient for calibration purposes. The applied technique of re-registration of controlled mis-registrations to evaluate the performance of registration algorithm has been used in several studies [28], [36]. This technique assesses the robustness of the registration algorithm, thereby giving a realistic idea on the accuracy that can be obtained.

The automated spatial normalization procedure (12-parameter affine) integrated in SPM8 showed registration errors within the range of 0.28 mm –0.59 mm and 0.98 mm –1.11 mm for [^18^F]FDG and [^18^F]FECT, respectively. These registration errors are in relative agreement with human literature data when considering relative values with respect to imaging resolution (e.g. corresponding to 2–3 mm accuracy for 4–10 mm resolution PET or SPECT devices) [37]. They are however higher than the values we reported ourselves for the rat brain, i.e. 0.24 mm –0.86 mm, using similar PET probabilistic atlases, probably because the number of voxels that encompass the rat brain is bigger than that of the mouse brain [28]. This mouse registration accuracy will likely be even better when using state-of-the-art small-animal PET systems that are characterized by a 5-fold volume resolution improvement [38].

Few reports have studied the implications of misalignments on quantitative measures in the (human) brain. Sychra et al. found that registration errors for single-axis-shift differences of 0.7 mm and 1° of rotation may produce intensity errors of 5%–10% on the voxel level using a voxelsize of 5.6 mm [39]. However, this estimation produces a maximum possible error that is diminished by considering larger adjacent regions or clusters as with the SPM approach where smoothing is the standard applied technique to level out intersubject anatomical differences in the spatial normalisation method. Therefore, it is expected that VOI analysis and statistical voxel-based analysis will result in significantly lower (semi)quantitative errors.

The mean translation, rotation, linear stretching and combination errors found in this work were strongly dependent on the radioligand used. The global cerebral uptake of [^18^F]FDG proved to yield superior registration accuracy as compared to [^18^F]FECT. [^18^F]FECT uptake is characterized by a very high signal-to-noise ratio and poor anatomical information due to the very high specificity of the radioligand.

The constructed probabilistic PET atlases were averaged from 10 genetically identical, adult, male C57BL6 mouse brains. Due to reported small structural volume- and surface area variabilities [10], they may also be used for spatial normalization of female gender and similar strains [8], although the uptake values or binding indices might theoretically slightly differ due to changes in the menstrual cycle or other hormonal effects. These mouse templates will be non-commercially made available for the small-animal molecular imaging community.

### Conclusion

In conclusion, functional mouse brain templates in stereotactic Paxinos co-ordinate space allow automatic anatomical standardization of anatomical and functional mouse brain scans with the necessary registration accuracy and the standardization of pre-defined VOI or SPM analysis. As test-retest and intersubject variability are comparable to the human situation, this forms the path for precise interventional or longitudinal studies in the mouse brain for disease characterization or treatment response.

## Supporting Information

Figure S1 Regression analysis of regional [^18^F]FDG uptake constant, K*FDG (a), and regional [^18^F]FDG rate constants, K1/k2 (b), k3 (c) and k4 (d), estimated by plasma and liver input functions.
(TIF)
