# Supplementary figures and images for: Construction and Evaluation of Quantitative Small-Animal PET Probabilistic Atlases for [18F]FDG and [18F]FECT Functional Mapping of the Mouse Brain
Source: PLoS One. 2013 Jun 7;8(6):e65286. doi: 10.1371/journal.pone.0065286 (PMC3676471; doi:10.1371/journal.pone.0065286)

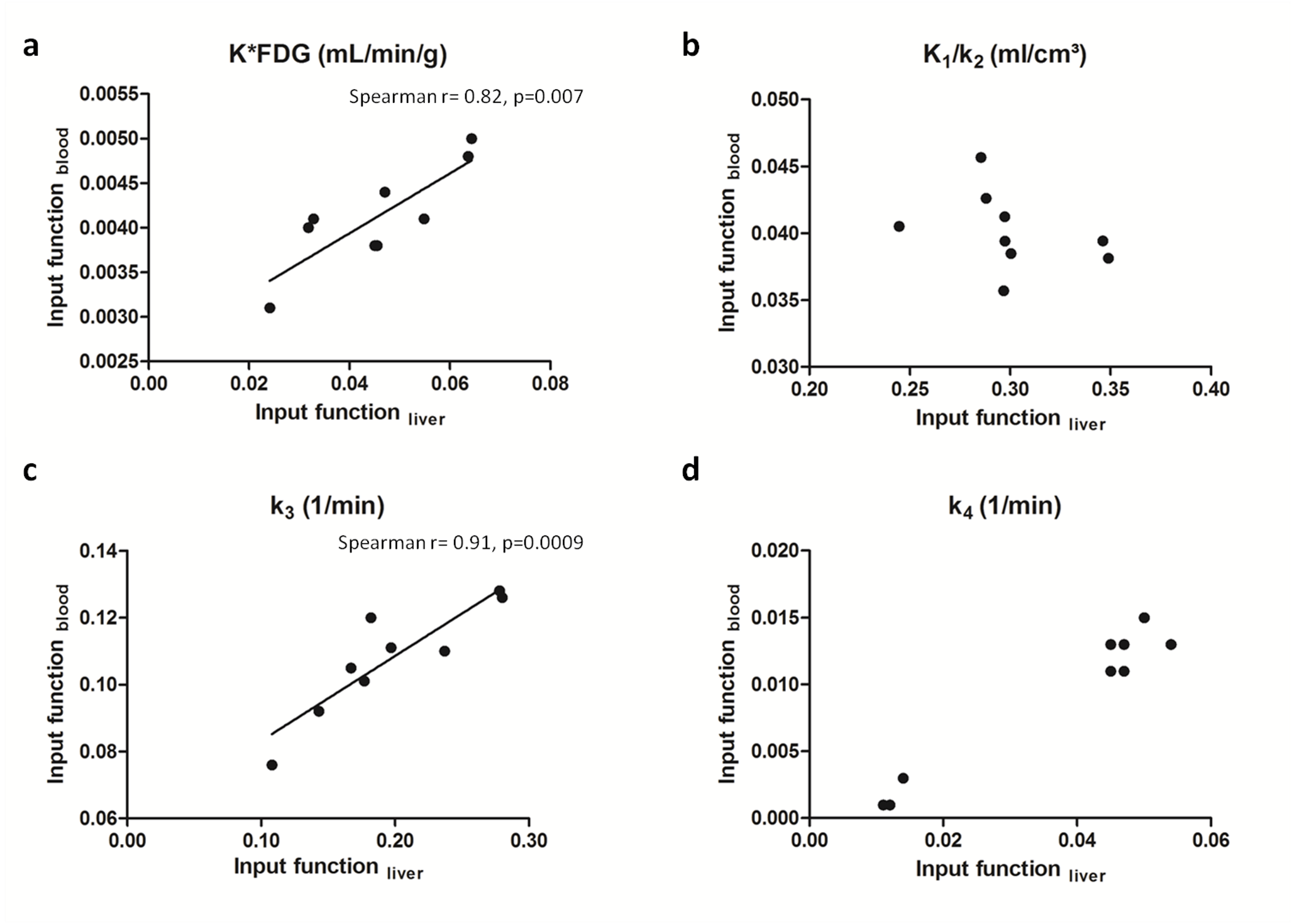

Supplement: Figure S1 — Regression analysis of regional [18F]FDG uptake constant, K*FDG (a), and regional [18F]FDG rate constants, K1/k2 (b), k3 (c) and k4 (d), estimated by plasma and liver input functions. (TIF) [file pone.0065286.s001.tif]
